# Krisengeprüftes Europa — auf dem Weg zu einer Patchwork-Union?

**DOI:** 10.1007/s10273-022-3112-9

**Published:** 2022-02-19

**Authors:** Heinz Handler

**Affiliations:** Wirtschaftspolitik, Technische Universität Wien, Karlsplatz 13, 1040 Wien, Österreich

**Keywords:** F15, F45, N14

## Abstract

Auf der Grundlage einer unzureichend gefestigten „europäischen Identität“ sah sich die Europäische Union in den vergangenen Jahren etlichen Herausforderungen gegenüber: der Globalisierung, der Finanzkrise, der Migration und dem populistischen Nationalismus, dem Brexit sowie der Coronapandemie. Die negativen Folgen konnten bis zu einem gewissen Grad aufgefangen werden. Die Auswirkungen des Klimawandels werden dies aber wahrscheinlich in den Schatten stellen. Die vorliegende Analyse unterstreicht den Mehrwert der europäischen Integration, dämpft aber auch die Illusionen über die Zukunft der EU. Obwohl die Krisen die Solidarität im Kern der EU gestärkt zu haben scheinen, sind auch die Unterschiede zwischen den Mitgliedstaaten deutlicher geworden. Das Ziel einer vollwertigen politischen Union ist in weite Ferne gerückt, und es lohnt sich daher, nach zweitbesten Lösungen zu suchen, wie etwa einer Form der differenzierten weiteren Integration.

## Abstract (en)

Based on an insufficiently consolidated “European identity”, the European Union has recently been exposed to the challenges of globalisation, financial disturbances, migration and populistic nationalism, Brexit, and the COVID 19 virus. The negative consequences of these somehow interrelated phenomena have to some extent been kept in check but are likely to be dwarfed by the looming threat of climate change. The present analysis underlines the added value of European integration, but also dampens illusions about the future of the Union. Although the crises seem to have strengthened the solidarity among the core of the EU, differences between member states have also become more apparent, as exemplified by the Brexit. The goal of a full-fledged political union is now a long way off, and it is therefore worth aiming for secondbest solutions, such as some form of differentiated further integration. The ongoing Conference on the Future of Europe offers the opportunity for an evaluation of such concepts.

Seit Ende des Zweiten Weltkriegs hat Europa eine enorme Wiederaufbau- und Einigungsperiode erlebt. Sie hat den politischen und wirtschaftlichen Bedeutungsverlust des Kontinents, der im Zuge der beiden Weltkriege eingetretenen war, wettgemacht. Im Kern ist diese Konsolidierung der frühen Beilegung französisch-deutscher Antagonismen zu danken, woraus die Integrationsbewegung entsprang, die in der heutigen EU ihren Höhepunkt erreicht hat. Der Integrationsprozess war noch zu Beginn des 21. Jahrhunderts von der Euroeinführung und der Osterweiterung geprägt. Dann folgten aber die weltweite Finanzkrise (2008/2009), die Eurokrise (2010 bis 2012), die Flüchtlings- und Migrationskrise (2015/2016), die Schritte zum Brexit (2016 bis 2020) und die sich über die Welt ausbreitende Coronakrise ab 2020. Gegenwärtig kämpft die EU – wie auch die übrige Welt – zudem gegen den fortschreitenden Klimawandel an.

Als Folge dieser Phänomene hat sich in der EU schrittweise eine Identitätskrise aufgebaut, die zeigt, dass das Integrationsgebilde nicht auf einem unverrückbaren Fundament steht, sondern laufend gerechtfertigt und an neue Entwicklungen angepasst werden muss. Die sukzessiv steigende Zahl der Mitgliedstaaten von ursprünglich sechs auf bis zu 28 hat die Heterogenität des politischen Meinungsspektrums und der Wirtschaftsstrukturen so weit erhöht, dass das umspannende Integrationsband zu reißen droht. Eine schmerzliche Folge war der Brexit, das Ausscheiden Großbritanniens aus der EU im Januar 2020. Damit ist die globale Bedeutung der EU als führender Wirtschaftsblock und als politischer Widerpart gegen US-amerikanische, russische oder chinesische Machtinteressen geschrumpft.

## Eurokrise bewältigt – Wachstumsziel verfehlt

Die globale Finanzkrise von 2008/2009 hat die EU zunächst als externe Herausforderung erreicht, um sich dann ab 2010 als Eurokrise zu einem internen Problem zu verdichten. Zur Bewältigung der Krise wurde ab 2012 eine Austeritätspolitik verfolgt, die von Befürwortenden als unumgänglich erachtet und von Gegner:innen als „neoliberale“ Fehlkonstruktion mit unabsehbaren wirtschaftlichen und gesellschaftlichen Schäden eingestuft wurde. Tatsächlich war die fiskalische Bremse für manche Problemländer eine unumgängliche Sofortmaßnahme, um

* Diesem Aufsatz liegt das soeben im Springer-Verlag erschienene Buch des Autors „Krisengeprüftes Europa: Wie wir die Solidarität in der EU stärken können“ zugrunde (https://www.springer.com/de/book/9783658352394).

**Dr. Heinz Handler** ist Dozent für Wirtschaftspolitik an der Technischen Universität Wien und stellvertretender Direktor der Europaplattform Wien-Brüssel.

Abbildung 1

**Wirtschaftsentwicklung in Deutschland und in den Programmländern des Euro-Rettungsschirms**

reales BIP pro Kopf

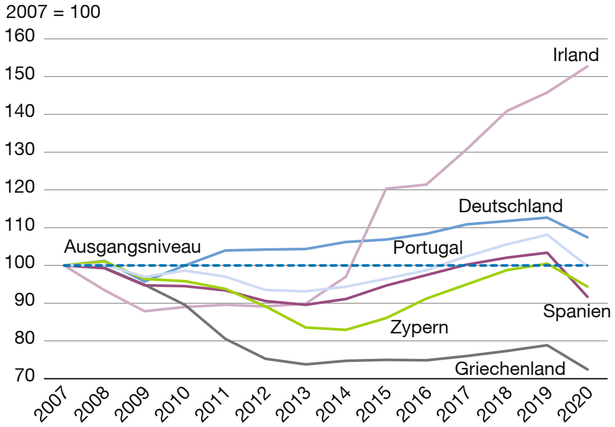

Quelle: Eurostat (2021).

Abbildung 2

**EU verfehlt wirtschaftliche Annäherung an USA**

reales BIP pro Kopf, Kaufkraftparitäten in internationalem US-Dollar – logarithmische Skala

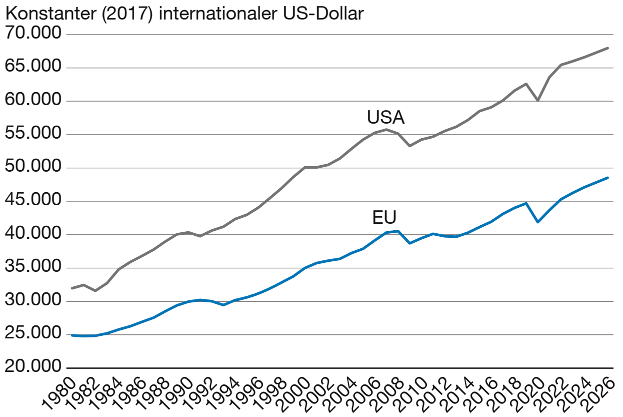

Quelle: Internationaler Währungsfonds (2021).

das Vertrauen der Finanzmärkte in die Zahlungsfähigkeit dieser Länder aufrechtzuhalten. Als Dauerlösung war sie nicht geeignet. Daher konnten die von der Eurogruppe (unter Mitwirkung des Internationalen Währungsfonds) geschnürten Rettungspakete, die auch das Schicksal der Gläubigerbanken im Auge hatten, nur eine befristete Unterstützung gewähren, deren Folgen und Erfolge in den betroffenen Programmländern durchaus unterschiedlich waren (vgl. Abbildung 1): Gemessen an der Entwicklung des realen BIP/Kopf waren Griechenland, Spanien und Irland von den Maßnahmen kurzfristig am stärksten betroffen. Allerdings fiel Irland von einem vergleichsweise hohen Einkommensniveau zurück und erholte sich spektakulär, wogegen das viel weniger reiche Griechenland weit zurückgeblieben ist. Im Jahr 2019 (also vor der Coronakrise) lag das BIP/Kopf in Griechenland immer noch um 21 % unter dem Niveau von 2007, in Zypern erreichte es gerade wieder den Vorkrisenwert. Unterschiedliche Zuwächse gab es in Spanien (+3 %), Portugal (+8 %) und Irland (+46 %). In Deutschland lag das BIP/Kopf um fast 13 % über dem Ausgangswert von 2007.

Ungeachtet der fiskalischen Austerität hat die Eurokrise dem europäischen Integrationsprozess wichtige Impulse gegeben: Es kam zur Errichtung des Europäischen Finanzaufsichtssystems (2011), zur Gründung des Europäischen Stabilitätsmechanismus ESM (2012), zum schrittweisen Aufbau einer Bankenunion (2014) und zu ersten Ansätzen für eine Kapitalmarktunion (2015). Dennoch hat die EU ihre selbstgesteckten Ziele nicht erreicht. Vor allem die propagierte Aufholjagd zum Einkommensniveau der USA ist gescheitert (vgl. Abbildung 2). Dies erfordert Anpassungen entweder bei den Zielen, indem man die Zielfunktion vom Pro-Kopf-Einkommen auf die gesamte Wohlfahrt inklusive Klimaziele ausdehnt (hier ist die EU nicht zu schlagen!) oder bei den operativen Instrumenten (Forcieren von Wachstumsträgern, die oft nicht mit den Klimazielen vereinbar sind) oder beim Verfassungsrahmen (auch auf mittlere Sicht unrealistisch).

## Unvollständig gebliebene Europaidee

Haben die mehr als ein halbes Jahrhundert anhaltenden Integrationsbemühungen in Europa eine kollektive europäische Identität hervorgebracht? Dafür sprechen die rapide Erweiterung der EU an Mitgliedsländern und die damit verbundenen wirtschaftlichen Vorteile, dagegen die unvermeidliche Zunahme an Heterogenität. Bei allen faktenbasierten Vorteilen der europäischen Integration ist diese gerade in Krisenzeiten einem erheblichen Gegenwind ausgesetzt.

Der Begriff der „europäischen Identität“ scheint offiziell erstmals in den Dokumenten für den Gipfel der EG-Staatsoberhäupter in Kopenhagen vom Dezember 1973 auf, in denen das „gemeinsame Erbe“ und die „eigenen Interessen“ der damals neun Mitgliedstaaten betont wurden. Mit „europäischer Identität“ war zu dieser Zeit eine positive Sichtweise verbunden, die ganz unterschiedliche Perspektiven des Integrationsprozesses abdeckte und ihren Ausdruck im (2000 eingeführten) Motto der EU „In Vielfalt geeint“ fand.

Das Verhältnis zwischen Vielfalt und Einheit ist über die Zeit nicht konstant geblieben. In Krisenzeiten nimmt die Bedeutung zentralstaatlicher Elemente regelmäßig zu

– wie etwa in der Eurokrise und in der Coronakrise. Als Ausnahme von dieser Regel gewannen bzw. behielten in der Migrationskrise die Nationalismen die Oberhand. Mittelfristig gibt es eher Weiterentwicklungen auf zwischenstaatlicher Ebene denn auf Gemeinschaftsebene. Für den früheren Präsidenten der Eurozone, Jeroen Dijsselbloem (2018), liegt dies im Wesen der EU, in der die Mitgliedstaaten über Vertragsänderungen sowie über neue Steuern und ihre Verwendung entscheiden. Dies gilt insbesondere auch für Krisenzeiten, in denen nicht ohne die Finanzkraft der Mitgliedstaaten und die Bereitschaft zur Zusammenarbeit vorgesorgt werden kann.

Die noch immer unvollendete europäische Identität ist als Antithese zu den Ideen der europakritischen radikalen Rechtspopulisten zu sehen, die sich nur der nationalen Identität als emotionaler Basis der Gesellschaft verbunden fühlen. Seit der Migration im Jahr 2015 erhalten diese Gruppen vermehrt Zuspruch, indem sie plakativ gegen die Bedrohung Europas durch massive Zuwanderung auftreten. Mit der Immigration kämen nicht nur europafremde kulturelle und religiöse Einflüsse ins Land, es würden damit auch Arbeitsplätze gefährdet. Nur der Nationalstaat könne effektiv dagegen vorgehen, die asylfreundliche EU verliere sich hingegen in fruchtlosen Debatten über die Verteilung und Integration von Geflüchteten. Faktum ist allerdings, dass nur im Rahmen der EU sowohl ein koordinierter Schutz der Außengrenzen als auch eine faire Verteilung der Lasten aus der erhöhten Migrationsbewegungen bewerkstelligt werden kann.

### Konstruktionsfehler stehen dem Integrationsziel der EU im Wege

In Umfragen wird die wirtschaftliche Integration nach wie vor als klarer Vorteil bekundet, doch gewinnen Gegenströmungen mit ausgeprägt nationalistischen Tendenzen an Stärke. Dies ist nicht nur eine Folge der Flüchtlingskrise, verschiedene große und kleine Konstruktionsfehler der EU laden die nationalen und regionalen Identitäten mit europakritischen Tönen auf und erschweren das Entstehen einer übergreifenden Europaidee.

Seit vielen Jahren wird versucht, einen eklatanten Mangel im Integrationsgefüge der EU zu beheben, jenen in der Sozialpolitik. Die schwerpunktmäßig bei den Mitgliedstaaten angesiedelten sozialen Agenden wurden durch den Vertrag von Amsterdam (1997/1999) in den primären Rechtsbestand der EU aufgenommen (Artikel 151 AEUV) und im November 2017 durch die „Europäische Säule sozialer Rechte“ konkretisiert. Dennoch ist die Asymmetrie zwischen der gut ausgebauten Politik zur Vermeidung von makroökonomischen Ungleichgewichten und den unzureichenden Koordinationsmechanismen im sozialen Bereich weiterhin gegeben (Aiginger und Kreuz, 2021). Zu den am häufigsten angeführten Unzulänglichkeiten gehört das Demokratiedefizit der EU-Institutionen. Es äußert sich schon im Mangel an gesamteuropäisch agierenden politischen Parteien, weshalb die Probleme der Gemeinschaft primär aus nationaler Sicht dargestellt und beurteilt werden. Die Missachtung des Spitzenkandidatenmodells für die Bestellung der Europäischen Kommission im Jahr 2019 durch die Mitgliedstaaten hat diesen Eindruck noch verstärkt. Beklagt wird auch die gefühlte Überregulierung durch „Brüssel“, selbst wenn die Verantwortung dafür meist bei den Mitgliedstaaten zu finden ist. Insgesamt wird die EU oft als „Projekt der Eliten“ gebrandmarkt, bei dem „das Volk“ nur wenig mitzureden habe.

Die Weiterentwicklung der EU als Gemeinschaft wird durch das Einstimmigkeitsprinzip im Rat behindert, das jedem Mitgliedstaat in grundsätzlichen Fragen eine Vetomöglichkeit einräumt. Im Gründungsvertrag der EWG war dagegen vorgesehen, diese Regel ab 1966 durch eine qualifizierte Mehrheit zu ersetzen, das ist aber im entscheidenden Moment am französischen Veto gescheitert. Heute müsste – angesichts der inzwischen sehr großen Zahl der Mitgliedstaaten – die Einstimmigkeit auf ganz wenige Grundsatzfragen eingeschränkt und jedenfalls in rein operativen Bereichen und in Finanzierungsfragen beseitigt werden. Als Systemmangel ebenso schwer wiegt der undurchsichtige Kompetenzdschungel zwischen den Mitgliedstaaten und der EU-Ebene sowie die verschwommene Aufgabenteilung auf EU-Ebene zwischen legislativer und exekutiver Gewalt.

Obwohl die Aufklärung des 18. Jahrhunderts als wichtige intellektuelle Basis für die europäische Integration und ihr Wertesystem gilt, hat sich deren Idee einer universellen Staatsbürgerschaft in der EU bisher nicht durchgesetzt. Die Unionsbürgerschaft (sie soll die nationale Staatsbürgerschaft nicht ersetzen, sondern ergänzen) wurde zwar 1992 durch den Vertrag von Maastricht eingeführt, sie hat aber bisher kaum identitätsstiftend gewirkt.

In der Wirtschaftspolitik der Eurozone hat sich die Dichotomie zwischen einheitlicher Geldpolitik und nationalstaatlichen Fiskalpolitiken zum einzig konsensfähigen Steuerungssystem entwickelt. In Krisenzeiten entstehen daraus enorme Friktionen zwischen Mitgliedsländern mit hoher Staatsverschuldung und hohen Kosten der Finanzierung auf der einen Seite und den Vertreter:innen einer nachhaltigen Austeritätspolitik auf der anderen. Die im Zuge der Coronapandemie aufgebaute Solidarität hat diese Spannungen etwas verringert, indem nun (zumindest temporär) auch gemeinschaftliche Finanzierungsmodelle diskutiert werden können.

## Kein Konsens in der Migrationspolitik

Im Inneren der EU hat sich das Spektrum politischer Ziele durch die erhöhte Migration ab 2015 dramatisch verengt, um nur noch einen Problembereich im Auge zu haben: die in manchen Augen eintretende Überfremdung. Dazu hat nicht nur die Zuwanderung selbst beigetragen, sondern auch das populistische Schüren von Ängsten für rein parteipolitische Zwecke. Verschwörungsmythen verbreiten sich rascher und nachhaltiger als die weniger sensationellen Tatsachen des Alltags. Europa wurde von der Zuwanderung unvorbereitet getroffen und besaß keine Strukturen zu dessen Bewältigung. Die hastig getroffenen Maßnahmen (z. B. Schließen der Balkanroute) überforderten die Asylsysteme der Anlandestaaten, sie konnten die irreguläre Migration nicht stoppen.

In allen betroffenen Ländern bewegt sich seitdem das politische Spektrum vom Zentrum weit nach rechts zu einer ausgeprägten Abwehrhaltung gegen Zuwanderung, auch gegen die von der Menschenrechtskonvention geschützten Geflüchtete. Der Zuwachs an Geflüchteten 2021 hat verdeutlicht, dass auf europäischer Ebene die Sicherung der Außengrenzen vorangetrieben werden müsste. Dieses Vorhaben kommt wegen abweichender Interessen der Mitgliedstaaten nicht entscheidend weiter, weshalb einzelne Länder ihre Probleme gegen die Regeln des Binnenmarkts mit der (Wieder-)Errichtung nationaler Grenzen und Zäune zu lösen versuchen. Im aktuellen Konflikt mit Weißrussland, das Drittlands-Geflüchtete an die Grenze zu Polen verfrachtet und dort aussetzt, stehen auch die Menschenrechte in der EU auf dem Prüfstand.

## Rechtspopulismus als Anti-Bewegung

In der politischen Realität Europas wird Populismus als eine Bewegung gegen die herrschenden Gruppen verstanden, auch wenn diese demokratisch legitimiert sind. Der nationalistische Rechtspopulismus stemmt sich gegen jede Überlagerung der nationalen Identität durch supranationale Elemente. Die Wahlerfolge der Rechtspopulisten lassen sich hauptsächlich auf Ängste vor einer schleichenden Änderung der kulturellen Werteordnung (Familienstrukturen, Religiosität) zurückführen, die kurz- bis mittelfristig noch von wirtschaftlichen Schocks überlagert sein können (Rodrik, 2019). Aktuell löst die Einwanderung von Personen mit niedriger beruflicher Qualifikation aus anderen Kulturkreisen Aversionen bei Teilen der Bevölkerung aus und fördert deren Unterstützung rechtspopulistischer bis rechtsextremer Akteure. Die beiden Gruppierungen unterscheiden sich in der Art des Gegners: Beim Rechtspopulismus ist es die Elite, beim rechtsextremen Nativismus sind es die ethnischen Minderheiten. Beide lehnen aber die EU in ihrer derzeitigen Form ab, weil sie

Abbildung 3
**Eurobarometer-Einschätzungen der wichtigsten EU-Probleme**

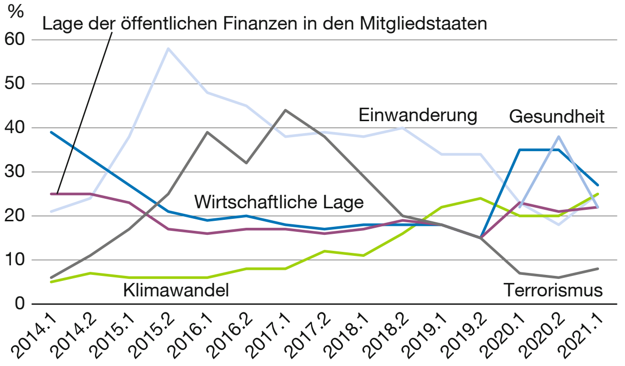

Quelle: Eurobarometer (2021).

Einschränkungen der nationalen Souveränität und Identität nicht hinnehmen wollen.

Die Migration wurde zwischen 2015 und 2019 in Eurobarometer-Umfragen meist als das größte Problem der EU bezeichnet. Erst die Pandemie hat die Ängste um die Gesundheit und die wirtschaftliche Lage in den Vordergrund gerückt, die Einwanderungsthematik ist aber im Sommer 2021 wieder verstärkt ins Bewusstsein der Befragten zurückgekehrt (vgl. Abbildung 3).

Die rechtspopulistischen und rechtsextremen Bewegungen verstecken ihren autoritären Kern häufig hinter dem Anspruch, im Interesse des „Volkes“ zu agieren. Der in Princeton tätige deutsche Politologe Jan-Werner Müller (2016) sieht aber im Rechtspopulismus nicht so sehr einen Kampf gegen die Eliten als vielmehr gegen Pluralismus. Daraus ergibt sich wiederum eine Gegnerschaft zur liberalen Demokratie, denn diese ist ohne Pluralismus nicht denkbar. Auch wenn rechtspopulistische Bewegungen die liberale Demokratie infrage stellen, sollte ihnen nach Taggart (2012) nicht mit einer Dämonisierung begegnet werden. Vielmehr müssten die von Populisten aufgegriffenen Problembereiche auch Gegenstand liberal-demokratischer Politik sein, die Antworten auf Fragen der Immigration, des Multikulturalismus und der weiteren europäischen Integration anzubieten hätte.

## Brexit: Die EU auf dem Weg zur Konsolidierung?

Der Austritt von Großbritannien aus der EU ist die Folge einer populistisch geführten innenpolitischen Auseinandersetzung, die keine Rücksicht auf die negativen ökonomischen Konsequenzen sowohl für Großbritannien als auch für die EU27 nahm. Zusätzlich bediente das Brexit-Referendum eine Reihe von Aversionen (gegen den europäischen Kontinent, gegen Immigrierende aus Polen) und Mythen (über ein – längst vergangenes – britisches Imperium).

Die Hintergründe des Brexits lassen sich anhand des Globalisierungstrilemmas von Rodrik (2007) ausleuchten. Dort wird die Unvereinbarkeit von Nationalstaat, Demokratie und Globalisierung in den Raum gestellt. Sampson (2017) greift diesen Gedanken auf und interpretiert den Brexit als demokratische Reaktion auf den schleichenden Verlust an britischer Souveränität im Zuge der fortschreitenden Integration in die EU. Für die EU bedeutet das umgekehrt, dass eine immer tiefere Integration der Mitgliedstaaten mit dem Fortbestand von nationaler Demokratie im Widerspruch zu stehen scheint. Daraus folgert Sampson für ein demokratisches Europa, dass sich entweder eine kollektive europäische Identität (anstelle der bisherigen nationalen Identitäten) herausbilden müsste oder die supranationale Komponente der EU zu reduzieren sein wird (indem man beispielsweise die Personenfreizügigkeit einschränkt). Ansonsten sei mit weiteren Austritten aus der EU zu rechnen.

Nach dem Brexit ist die zurückbleibende EU27 weniger heterogen als zuvor. Gesamteuropäisch hat aber die Solidarität gelitten, und es ist nicht auszuschließen, dass sich sowohl bei den Briten (Schottland, Nordirland) als auch in der EU (Katalonien, Flandern) manche Separatismen verfestigen. Vom Brexit sollte die EU lernen, dass ein Gleichklang bei den Grundwerten wichtiger ist als vordergründige Größe.

Empirische Studien zu den makroökonomischen Folgen des Brexit ergeben, dass auf beiden Seiten des Kanals Wohlfahrtsverluste zu verbuchen sind, die aber in Großbritannien höher ausfallen als in der EU27. Gravierende Verwerfungen sind allerdings auszuschließen, weil einerseits nun schon mehrere Jahre für Anpassungen genutzt werden konnten und andererseits mit dem neuen Handelsabkommen nicht alle Brücken zur EU abgebrochen werden. Offen ist allerdings, ob das Abkommen hinsichtlich Nordirland voll umgesetzt werden kann.

### COVID-19 als Test für Solidarität

Eine gewisse Unterstützung für die europäische Solidarität kam von der Coronapandemie, deren gesundheitliche, wirtschaftliche und soziale Schäden dem Gemeinschaftsgedanken einen unverhofften Impuls versetzt haben. Gleichzeitig haben die Ängste vor Krankheit und/oder Impfung sowie das holprige Anlaufen von nationalen und gemeinschaftlichen Maßnahmen ein neues Betätigungsfeld für Rechtspopulisten und ihre Verschwörungsmythen eröffnet. Maskenverweigernde, Lockdown- und Impfgegner:innen können sich mit jenen verbünden, die durch die Krise in gesundheitliche, psychische oder wirtschaftliche Schwierigkeiten geraten sind.

Welche Rolle konnte die EU ohne zentrale Kompetenzen im Gesundheitsbereich bei der Bewältigung der Pandemie spielen? Wurden die wirtschaftlichen und politischen Folgen der Pandemie optimal verkraftet? Als sich die Coronapandemie auszubreiten begann, konnte die schon seit der Migrationskrise eingeübte Abschottung der Binnengrenzen aktiviert werden. Damit ließ sich zwar die Ausbreitung des COVID-19-Virus nicht verhindern, wohl aber wurden nationale Schutzmaßnahmen ohne langwierige Abstimmung auf EU-Ebene rasch verwirklicht. Als Kehrseite konnte Frankreich Anfang März 2020 kurzfristig alle Atemschutzmasken im Lande beschlagnahmen und Deutschland – entgegen aller Binnenmarktregeln – eine bereits eingeleitete Lieferung von medizinischer Schutzausrüstung nach Frankreich verhindern.

Mittel- bis langfristig wird die Coronakrise wohl auch eine Neubewertung der globalen Wertschöpfungsketten in Gang setzen, die auch eine Sicherung von „strategischen“ Produktionen in der EU zum Ziel hat. Auf diesem Weg sollten aber weder die nationalen Protektionismen verstärkt noch essenzielle Produktionen in Entwicklungsländern (weiter) unterwandert werden (z. B. über Exportsubventionen für die EU-Landwirtschaft).

Die EU wurde von der Coronakrise zu einem Zeitpunkt getroffen, als sie noch in der Bewältigung der Vorgängerkrisen verhaftet war und die neue EU-Kommission ihre Pläne in Umsetzung bringen wollte. Einige Aspekte der Pandemie erinnern an die zuvor gerade überwundenen Krisen. An die Flüchtlingskrise gemahnen die Lockdowns und Grenzschließungen, an die Eurokrise die enormen staatlichen Hilfen und ihre Finanzierung. Ein wichtiger Unterschied zur Finanzkrise ist, dass damals wirtschaftliche Ungleichgewichte als Auslöser fungierten, während sie nun eine Folge der Krise sind. Im Unterschied zur Flüchtlingskrise, die in Zahlen gemessen werden konnte, bestand nun die Gefahr eines sich schleichend anbahnenden Kollapses des Gesundheitssystems.

Manche der über Jahre entstandenen politischen und wirtschaftlichen Risse zwischen den EU-Ländern wurden durch den Schock über COVID-19 kurzfristig zugedeckt. Sie traten aber wieder hervor, als die ersten Notmaßnahmen die Pandemie einzudämmen schienen. Dazu kamen neue, durch die Coronakrise selbst ausgelöste Zwiste, etwa um die Zulassung und Verteilung von Impfstoffen oder um binnenmarktwidrige Grenzschließungen. Die Konfliktlösung kostete nicht nur Zeit, sondern zeigte (wieder einmal) die Grenzen für die Integrationsbereitschaft in Europa auf.

Zur Begrenzung der wirtschaftlichen Schäden der Pandemie waren auf nationaler und EU-Ebene enorme Mittel aufzuwenden. Die Politik stand vor der Herausforderung, die begrenzten Fördermittel auf die überlebenswichtigen und -fähigen Projekte zu konzentrieren. Die Pandemie breitete sich aus, als in der EU gerade das mittelfristige EU-Budget 2021-27 verhandelt wurde. Dazu kam nun das Erfordernis, auf EU-Ebene Finanzhilfen zu gewähren, um in Ergänzung zu den nationalen Anstrengungen einen Zusammenbruch ganzer Wirtschaftsbereiche zu verhindern.

Daraus entstand ein Paket aus zwei zentralen Komponenten, dem Mehrjährigen Finanzrahmen (MFR) 2021/27 und dem temporären Aufbauinstrument „Next Generation EU" (NGEU) mit einem Gesamtvolumen von 1.824,3 Mrd. Euro (zu Preisen von 2018). Die 750 Mrd. Euro des NGEU-Programms wird die EU langfristig (bis 2058) über gemeinschaftliche Schuldtitel mit solidarischer Haftung aller Mitgliedstaaten (Corona-Bonds) am Kapitalmarkt refinanzieren. Die Projekte werden in den Nationalen Aufbau- und Resilienzplänen (NARPs) definiert, die im Rahmen des Europäischen Semesters auf Basis der länderspezifischen Empfehlungen des Rates abgewickelt werden. Damit wird der Aufbau nach der Krise wiederum von den regionalen Sichtweisen der Mitgliedstaaten dominiert, gemeinschaftliche Ziele (abgesehen von den EU-weit vorgeschriebenen Projekten zur grünen Wirtschaft und zum digitalen Wandel) werden allenfalls bei der Finanzierung berücksichtigt. Dem übergeordneten Ziel wäre besser gedient gewesen, wäre die Auswahl der Projekte nach gemeinschaftlichen Gesichtspunkten von einem unabhängigen (dem Europäischen Parlament berichtspflichtigen) Gremium vorgenommen worden.

Völlig verabsäumt wurde es, bei dieser Gelegenheit auch gleich die schon lange anstehende Strukturbereinigung im EU-Budget anzugehen und sie mit einer Vertiefung des Subsidiaritätsprinzips zu verknüpfen. In der Kohäsionspolitik wäre dies ebenso möglich wie in der Agrarpolitik, hier etwa mit einer Verlagerung der „Säule 1" (Einkommensstützung und Marktmanagement) auf die nationale Ebene. Im Gegenzug müssten gemeinschaftsrelevante Fragen des Klimawandels, der Pandemiesteuerung und der menschenwürdigen Behandlung von Geflüchteten auf die EU-Ebene gehoben werden.

## Erfolgreiche Krisenstabilisierung

In Krisenzeiten erweist sich Solidarität als besonders gefragtes Verhalten. Viele Beobachter:innen bewerten daher die fiskalischen Maßnahmen der EU zur solidarischen Bewältigung der Coronakrise als beachtlichen Erfolg, weil der Nachfrage- und Produktionsausfall rasch stabilisiert werden konnte. Hingegen gibt es Kritik an der Allokationsfunktion der Maßnahmen. Das Programm NGEU ist mit der Vorgabe gestartet worden, maßgeblich zum Klimaschutz und zur Digitalisierung beizutragen. In der Umsetzung werden allerdings bei vielen Projekten Substitutionseffekte vermutet, indem schon länger vorbereitete nationale Vorhaben nun von der Gemeinschaft finanziert werden. Außerdem gibt es keine klare Trennung zwischen rein privaten Aktivitäten mit individueller Gewinnerwartung und jenen Aufgaben, die nur von der öffentlichen Hand erfüllt werden können und daher eine gemeinschaftliche Finanzierung rechtfertigen. Hinsichtlich der Verteilungsfunktion gewähren die Mittel aus NGEU nicht primär einen Ersatz für Schäden aus der Coronakrise. Vielmehr verteilen sie netto von reicheren zu ärmeren Ländern (gemessen am BIP/Kopf) um und ergänzen damit nur die bisherige Kohäsionspolitik (Fuest, 2021).

Bei aller Kritik an den komplexen Hilfsprogrammen muss man anerkennen, dass sich die EU und ihre Gremien in Krisenzeiten als ausreichend flexibel erwiesen haben, frühere rote Linien zu überschreiten, um die Wohlfahrt in der Gemeinschaft zu optimieren. Die Zukunft der EU wird auch daran zu messen sein, wie erfolgreich in der Coronakrise nationale Alleingänge den Gemeinschaftsinteressen untergeordnet werden konnten.

Um in der Coronakrise die Funktion der Banken als Finanzintermediärinnen und Risikotragende aufrecht zu halten, hat die Europäische Zentralbank seit dem Frühjahr 2020 eine Reihe von Lockerungsmaßnahmen beschlossen. Als Ergebnis dieser Politik ist die Liquidität im Bankensektor massiv angestiegen, sodass sie zur Jahresmitte 2021 um ein Vielfaches höher war als während der Eurokrise. Bei den privaten Nichtbanken hat sich dies in einer drastischen Erhöhung der Sparquote und in einer leichten Zunahme der Bargeldhaltung niedergeschlagen (vgl. Abbildung 4). Die expansive Geldpolitik hat insgesamt verhindert, dass die übrigen Hilfsmaßnahmen auf europäischer und nationaler Ebene an Finanzierungsgrenzen gestoßen wären. Die von vielen befürchtete Inflation beschränkt sich bisher auf exogene Faktoren, doch sind die Währungsbehörden gut beraten, eine sukzessive Rückführung der Überliquidität vorzubereiten.

Bei den Verhandlungen über die Finanzierung der Maßnahmen gegen die Coronakrise ist das schon längere Zeit schwelende Problem der Rechtsstaatlichkeit virulent geworden. Die Kommission hat insbesondere gegen Polen und Ungarn Verfahren nach Artikel 7 EUV eingeleitet, um Vorwürfe des Verstoßes gegen diesen Grundsatz der EU zu prüfen und allenfalls mit Sanktionen dagegen vorzugehen. Letztlich stellt sich die Frage, ob ein anhaltendes Abweichen eines Mitgliedstaats von etablierten Grundsätzen der EU nur über einen Austritt gelöst werden kann oder ob

Abbildung 4
**Liquiditätsindikatoren für den Euroraum**

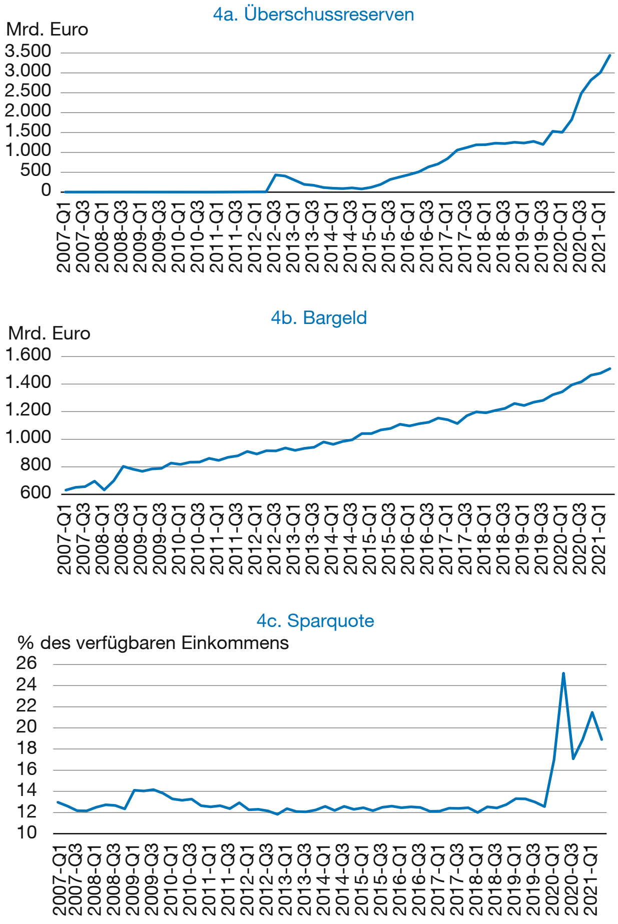

Quelle: Eurostat.

und in welcher Form Raum für eine Peripherie von Ländern mit eingeschränkten Rechten und Pflichten besteht.

Solche Überlegungen führen zu einem Kerneuropa-Modell mit differenzierter Integration. In diesem Sinne plädiert etwa Ohr (2017) dafür, an Stelle von „mehr Europa" ein „besseres Europa" anzustreben, das den Mitgliedstaaten mehr Flexibilität zugesteht. Nur wenn nicht immer alle Mitglieder mit ihren unterschiedlichen wirtschaftlichen und sozialen Rahmenbedingungen sämtliche Integrationsschritte mitmachen müssen, werden sie sich weiterhin mit der EU identifizieren können. Es gelte, die bisherigen Errungenschaften der europäischen Integration, insbesondere des europäischen Binnenmarkts, zu betonen und die Integrationsbereitschaft nicht durch Forderungen nach einer „immer engeren Union" aufs Spiel zu setzen.

## Welche Zukunft hat die EU?

Haben die wiederkehrenden Krisen den europäischen Integrationsprozess gestärkt oder geschwächt? Ist die EU auf dem Weg zu einer „differenzierten Integration" oder zu einer „differenzierten Desintegration" (Tekin, 2016), wenn nicht überhaupt zur Auflösung? Die Krisen im vergangenen Jahrzehnt haben die Grenzen einer weitgehend abstrakten Europaidee aufgezeigt. Da es gemeinschaftliche Institutionen derzeit nur in Ansätzen gibt, lässt sich „Europa" der Bevölkerung auf nationaler Ebene nur unzureichend näherbringen. Von den wahlabhängigen nationalen Politiker:innen sind keine Initiativen zur Beschränkung ihrer eigenen Position zu erwarten, weshalb es ohne äußeren Druck auf absehbare Zeit keine verfassungsmäßig abgesicherte entscheidende Stärkung der EU-Ebene geben wird.

Langfristig wird eine weitere Vertiefung des Integrationsziels der EU nur zu erreichen sein, wenn die demokratische Legitimation von EU-Institutionen gestärkt und die Gewaltenteilung besser verankert wird. Um die Wähler:innen ausreichend mit europäischen Fragestellungen und Standpunkten vertraut zu machen, bedarf es politischer Parteien, die über ihre regionalen und nationalen Fragestellungen hinaus auch europäisch argumentieren und werben können.

Um das bisher erreichte Integrationsniveau zu halten und mit Leben zu erfüllen, sollte dem in Artikel 5 Absatz 3 EUV festgelegten Subsidiaritätsprinzip konsequenter als bisher Rechnung getragen werden. Als Gegenleistung könnte von den Mitgliedstaaten mehr Solidarität bei gemeinschaftlichen Anliegen eingefordert werden. Im daraus resultierenden System wären gesamteuropäische Ziele auf der EU-Ebene (Top Down) zu definieren, die Umsetzung sollte aber auf nationaler und regionaler Ebene (Bottom Up) erfolgen, dort nämlich, wo der politische Kontakt zu den betroffenen Personen mit ihren Bedürfnissen laufend gepflegt wird. Die Kontrolle der Umsetzung müsste dann wieder einheitlich auf europäischer Ebene angesiedelt sein.

Die Kaskade von Krisen, denen die EU in den vergangenen eineinhalb Jahrzehnten ausgesetzt war, hat das Gefühl der Zusammengehörigkeit in Europa tendenziell gestärkt. Dies sollte für die „Konferenz zur Zukunft Europas" genutzt werden, die seit Mai 2021 an Vorschlägen zur Beseitigung der in den Krisen sichtbar gewordenen institutionellen Mängel der EU arbeitet (Europäische Kommission, 2021). Hier sollten die von den Skeptiker:innen der europäischen Integration aufgeworfenen Fragen und Anliegen aufgegriffen und verarbeitet werden. Ein solcher Reformanlauf könnte die EU auf einer verbesserten demokratischen Basis handlungsfähiger machen. Die EU könnte auf diese Weise zum Vorbild für das friedliche Miteinander unterschiedlicher Länder mit nicht deckungsgleichen Kulturen werden. Einen Beitrag zur Zukunft der EU wird diese Konferenz aber nur leisten können, wenn sie mit einem konsensfähigen Gesamtpaket den Reformwillen in den Mitgliedstaaten stärken kann.

Die EU ist eine hybride Staatsform mit dominierenden Elementen eines Staatenbundes und einigen abgegrenzten Bereichen eines Bundesstaats. Aufgrund eines Urteils des Deutschen Bundesverfassungsgerichts von 2009 ist die EU auch nach dem Vertrag von Lissabon „kein Bundesstaat, sondern bleibt ein Verbund souveräner Staaten unter Geltung des Prinzips der begrenzten Einzelermächtigung". Nicht nur rechtlich, auch politisch konnte der ursprüngliche Ansatz, als vereintes Europa in der Welt mit einer Stimme zu sprechen, bisher nicht verwirklicht werden. Im Verhältnis zur Welt ist Europa zwar wirtschaftlich eine Großmacht, politisch ist es aber nach wie vor zersplittert, sodass in internationalen Gremien (Vereinte Nationen, Internationaler Währungsfonds, Weltbank etc.) oft nur seine größeren Mitgliedstaaten wahrgenommen werden. Diese Schwäche hat mit dem Austritt Großbritanniens aus der EU noch zugenommen.

Die EU ist ein zutiefst politisches Projekt mit einem enormen wirtschaftlichen Potenzial, es ist allerdings noch nicht weit über seine wirtschaftlichen Anfänge hinausgekommen. Die Krisen haben das Integrationsprojekt der EU geprägt: Sie haben den Kern der EU gestärkt, gleichzeitig haben sie aber die Unterschiede zwischen den Mitgliedstaaten schärfer hervortreten lassen. Damit ist die Idee einer politischen Union als optimales Fernziel für den Kontinent in weite Ferne gerückt. Für die Zeit bis dahin lohnt es sich, zweitbeste Lösungen anzupeilen. Einige davon wurden hier diskutiert und Wege zu ihrer Verwirklichung aufgezeigt.

**Title:** ***Crisis-tested Europe – Towards a Patchwork Union?***
